# Hormonal and circuit mechanisms controlling female sexual behavior

**DOI:** 10.3389/fncir.2024.1409349

**Published:** 2024-05-01

**Authors:** Sayaka Inoue

**Affiliations:** Department of Psychiatry, School of Medicine, Washington University in St. Louis, St. Louis, MO, United States

**Keywords:** social behavior, ovarian hormones, female, sexual behavior, hypothalamus

## Abstract

Sexual behavior is crucial for reproduction in many animals. In many vertebrates, females exhibit sexual behavior only during a brief period surrounding ovulation. Over the decades, studies have identified the roles of ovarian sex hormones, which peak in levels around the time of ovulation, and the critical brain regions involved in the regulation of female sexual behavior. Modern technical innovations have enabled a deeper understanding of the neural circuit mechanisms controlling this behavior. In this review, I summarize our current knowledge and discuss the neural circuit mechanisms by which female sexual behavior occurs in association with the ovulatory phase of their cycle.

## Introduction

Sexual behavior is essential to reproduction in many animals and is instinctual that the behavior can be displayed without any prior experience. This instinct suggests that the behavior is genetically hard-wired, with the corresponding neural circuits being generated and established during the developmental phase. In many vertebrates, female sexual behavior is synchronized with the estrous cycle. Females are sexually receptive toward male mounting only during a short period surrounding ovulation (estrus phase) and are not receptive during the other stages of the estrous cycle. For example, female mice are sexually receptive once in every four to five days while female giant pandas are receptive only a few days in a year (Allen, 1922; Lindburg et al., 2001). Despite the variability in the duration of female estrous cycles across species, the concurrence of ovulation and female sexual receptivity is a common trait among many mammals. This synchronization is crucial for efficient reproduction, minimizing the risk of predation and waste of energy. Female sex hormones 17β-estradiol (referred to as estrogen here) and progesterone are released from the ovary and their levels peak at the timing surrounding ovulation. Over the decades, studies revealed that estrogen and progesterone, and their receptors, are essential to female sexual behavior (Ring and Providence, 1944; Powers, 1970; Beach, 1976; Lydon et al., 1995; Rissman et al., 1997; Arnold, 2009). Recent studies employing novel genetic, imaging, and behavioral approaches further characterized how these sex hormones contribute to the behavior by modulating neural circuits in the female brain. In this review, I illustrate the overview of how this alliance between ovulation and female sexual behavior is controlled by neural circuitry in the brain.

## Female sexual behavior depends on female steroid hormones

The sex steroid hormones estrogen and progesterone, produced and released by the ovary, are essential for inducing female sexual behavior. These sex hormone levels are closely related to the estrous cycle: they peak around the estrus, the stage that female is sexually receptive and ovulating, while it goes down to undetectable levels at the other stages of the estrous cycle (Deleon et al., 1990; Nelson et al., 1992). Removal of ovaries eliminates these changes in sex hormone levels, and consequently, female sexual receptivity. Subsequent supplementation of estrogen and progesterone rescues the behavior in the ovariectomized (OVX) female, demonstrating that female sexual behavior can be induced by increased levels of sex hormones without actual ovulation. This OVX and hormonal priming regimen is widely used to induce female sexual behavior in experimental animals (Whalen, 1974; Nelson et al., 1992; Lydon et al., 1995; Rissman et al., 1997; Ogawa et al., 1998; Bakker et al., 2002; Kudway and Rissman, 2003; Wu et al., 2009; Xu et al., 2012; Yang et al., 2013).

Estrogen and progesterone bind to their cognate receptors such as estrogen receptor a (ERa or Esr1) and progesterone receptor (PR). These receptors are known as transcription factors that they alter gene expression and protein synthesis. Studies utilizing knockout mice of the receptors revealed that signaling of these sex hormones in the brain is essential for female sexual behavior (Lubahn et al., 1993; Lydon et al., 1995; Rissman et al., 1997). Inhibition of protein synthesis in the brain also suppresses the behavior even when OVX females are supplemented with sex hormones (Rainbow et al., 1980, 1982; Meisel and Pfaff, 1985). Together, these findings suggest that a sequence of steroid hormone signaling, followed by gene expression and protein synthesis in the brain, is essential for inducing female sexual behavior.

A more recent study employing microarray, bulk, and single-nucleus RNA sequencing compared gene expression profiles in four different hypothalamic and limbic regions between OVX and hormonally primed OVX females (Xu et al., 2012; Knoedler et al., 2022). Notably, Knoedler et al. (2022) identified 1,415 genes whose expression changes in four limbic and hypothalamic regions depending on sex hormones. This is the first report to highlight such extensive changes in gene expression profiles due to different hormonal states in females. Which genes are critical for controlling female sexual behavior? Gene ontogeny analysis identified that they include genes in synaptic transmission, steroid hormone-related processes, behavior, peripheral reproductive organ processes, and regulation of gene expression. The expression levels of these hormone-sensitive genes vary across different brain regions. Therefore, to elucidate roles of these identified hormone-sensitive genes, it is critical not only to analyze brain-wide gene knockout animals but also to employ region-specific viral delivery of CRISPR-Cas9 for gene knockout and RNAi for gene knockdown (Musatov et al., 2006).

## Key neural circuits controlling female sexual behavior

**Figure 1:**
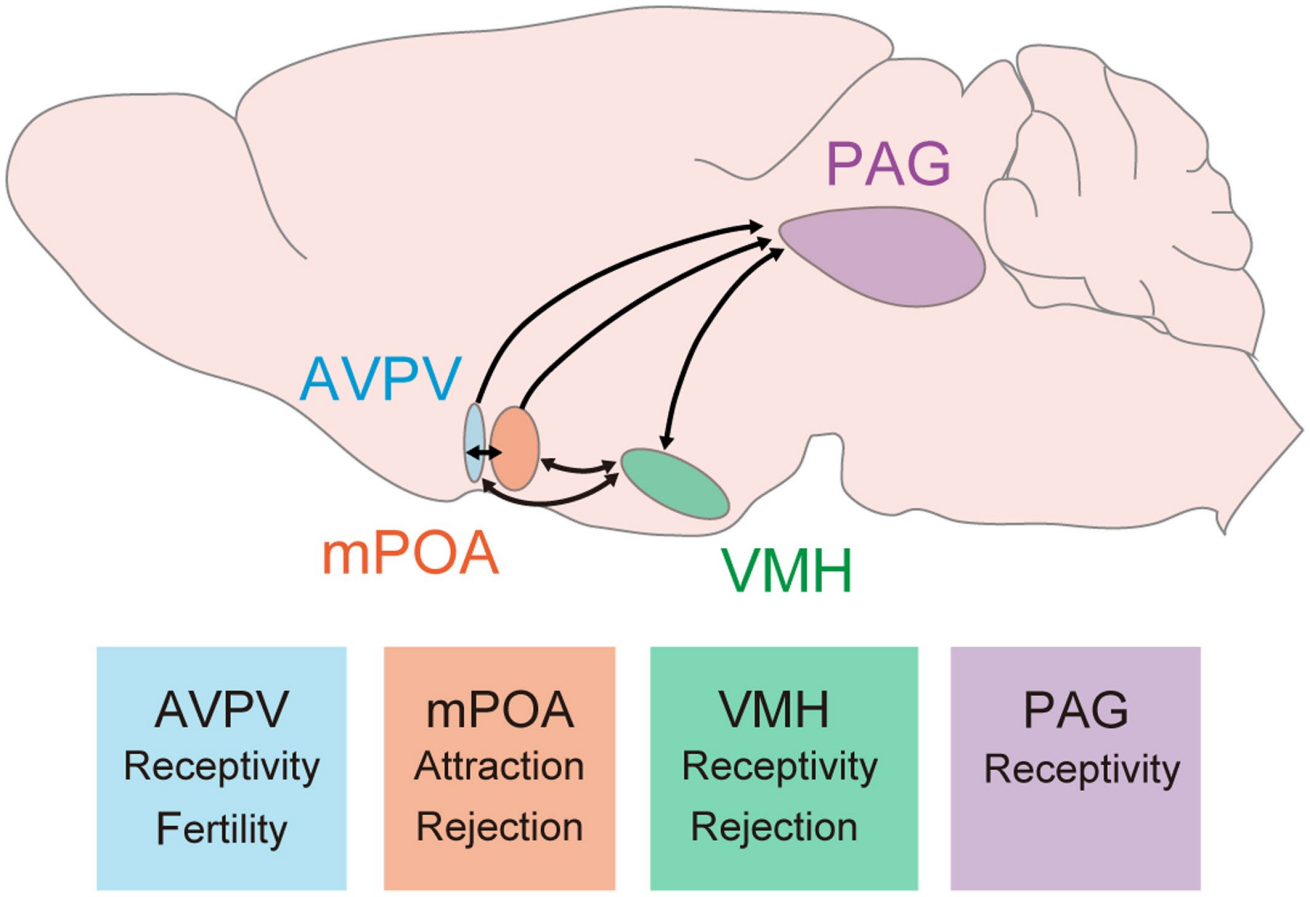
Key brain regions and their roles in female sexual behavior. These hypothalamic and midbrain circuits have been studied and demonstrated their contribution to female sexual behavior. Arrows indicate connections between these regions. It is important to note that reciprocal connections between the PAG and either the AVPV or mPOA have not yet been reported; however, it is possible that such connections exist. Major findings related to female sexual behavior are described at bottom boxes. AVPV, anteroventral periventricular nucleus; mPOA, medial preoptic area; VMH, ventromedial hypothalamus; PAG, periaqueductal gray.

Many hypothalamic and limbic regions in the brain are found to be involved in female sexual behavior. As key regions, the ventromedial hypothalamus (VMH), medial preoptic area (mPOA), and periaqueductal gray (PAG) were identified decades ago as they are critical to the behavior (Figure 1). The induction of the immediate early gene Fos in these regions during female sexual behavior suggests their activation (Pfaus et al., 1993; Blaustein et al., 1994; Flanagan-Cato and Mcewen, 1995; Yamada and Kawata, 2014). Importantly, histological studies indicate that these regions are rich in expression of sex hormonal receptors such as Esr1 or PR (Wu et al., 2009; Cheong et al., 2015; Kim et al., 2019). Local infusion of sex hormones into these areas modulates female sexual behavior, underscoring their control in association with the estrous cycle (Robert, 1962; Barfield and Chen, 1977; Floody et al., 1986; Rajendren et al., 1991). Early work by Sakuma and Pfaff in the 1970s highlighted the importance of these brain regions in modulating female sexual behavior. This review will summarize the roles of each circuit in detail.

**Figure 2:**
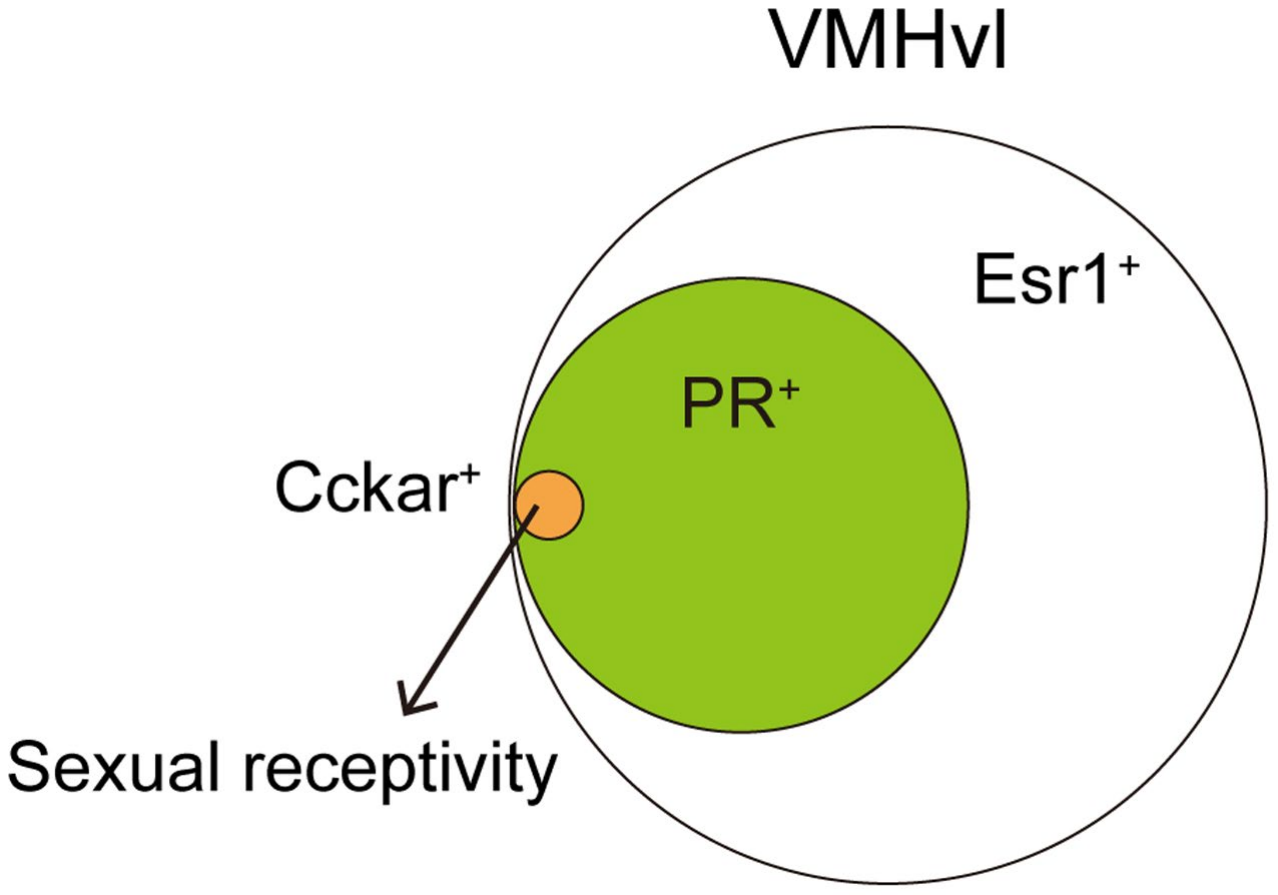
A small subset of VMHvl neurons can drive female sexual behavior. About 60% of the VMHvl neurons expressing Esr1 are also PR^+^. Further, Cckar^+^ population is one of 27 clusters identified in Esr1^+^ VMHvl neurons. Such a small subset of neurons is critical to drive female sexual behavior. Cckar^+^ VMHvl neurons, as well as PR^+^ VMHvl neurons, project to the AVPV and this connectivity change depending on ovarian hormone levels.

The VMH, especially the ventrolateral part of the VMH (VMHvl), is well characterized its role in female sexual behavior. Single-unit recordings of neuronal firing in awake mice and primates reveal increased firing frequency in VMHvl neurons during conspecific male investigation or upon receiving male mounting and intromission (Aou et al., 1988; Nomoto and Lima, 2015). Electrical stimulation of the VMHvl enhances, while lesions suppress, female sexual receptivity, indicating this region’s necessity and sufficiency (Pfaff and Sakuma, 1979a,b). Moreover, sex hormones play crucial roles in this modulation. Local hormone infusion in the VMHvl elevates female sexual behavior (Barfield and Chen, 1977; Rajendren et al., 1991). Histological studies demonstrate that expressions of sex hormonal receptors, such as Esr1 or PR, are dense in the VMHvl (Xu et al., 2012; Yang et al., 2013). Approximately 50% of VMHvl neurons are Esr1^+^, with these neurons becoming active during male–female interactions (Hashikawa et al., 2017). Knockdown of Esr1 gene in the VMHvl via RNAi decreases female sexual receptivity (Musatov et al., 2006), suggesting that Esr1 signaling in the VMHvl is necessary to the behavior. Recent studies have further delineated the neuronal populations in control. Almost all PR^+^ VMHvl neurons co-express Esr1, with about 60% of Esr1^+^ VMHvl neurons being PR^+^ (Yang et al., 2013). These PR^+^ VMHvl neurons, when active during male mounting and lordosis behavior, are crucial for driving female sexual behavior, as shown by fiber photometry imaging (Inoue et al., 2019). Genetically targeted ablation or acute chemogenetic inhibition of these PR^+^ VMHvl neurons reduces female sexual receptivity, even in hormonally primed OVX females (Yang et al., 2013; Inoue et al., 2019). These findings indicate that PR^+^ VMHvl neurons are essential to drive female sexual behavior. Single-nucleus RNA sequencing from Esr1^+^ VMHvl neurons identified a subset expressing the cholecystokinin-a receptor (Cckar) within total 27 of clusters (Figure 2), significant only in hormonally primed OVX females and not in males or OVX females, aligning with earlier microarray and *in situ* hybridization studies (Xu et al., 2012; Knoedler et al., 2022). This suggests the critical role of Cckar^+^ VMHvl neurons in female sexual behavior. Fiber photometry imaging revealed that Cckar^+^ neurons are active during mating, like PR^+^ VMHvl neurons (Yin et al., 2022). Further, behavioral studies with acute inhibition of Cckar^+^ VMHvl neurons, conducted by two different groups, found that these neurons are crucial to female sexual receptivity (Knoedler et al., 2022; Yin et al., 2022). Thus, Cckar^+^ VMHvl neurons are the subset of Esr1^+^ PR^+^ VMHvl neurons that control female sexual behavior. Gene knockout of Cckar abolishes female sexual receptivity (Xu et al., 2012), however, whether Cckar gene expression within these neurons is necessary remains to be elucidated. Considering the increased Cckar expression in OVX primed females, it is possible that both firing activity and gene expression in Cckar^+^ VMHvl neurons control the behavior in a coordinated manner. These Cckar^+^ VMHvl neurons exist mostly in the center to posterior VMHvl. More recent study indicates that anterior PR^+^ VMHvl neurons can drive rejection towards male mounts (Gutierrez-Castellanos et al., 2023). The detailed molecular identity of these anterior PR^+^ VMHvl neurons and connection between Cckar^+^ VMHvl neurons to regulate sexual receptivity and rejection are interesting topics to be revealed in future studies.

The PAG is one of the major downstream circuits receiving inputs from VMHvl. The PAG has been shown to increase lordosis upon electrical stimulation and decrease behavior upon lesioning, highlighting its role in modulating female sexual responsiveness (Sakuma and Pfaff, 1979a,b). Electrophysiological recordings from the PAG of anesthetized rats found that PAG neurons response to VMH and mPOA stimulation, suggesting that it works as the downstream of these neural circuits (Sakuma and Pfaff, 1980). The complexity and size of the PAG make it challenging to focus on specific subregions, which may explain the scarcity of *in vivo* electrophysiological recordings or calcium imaging studies specifically addressing female sexual behavior. Recent advances in spatial transcriptomics have identified distinct neural populations within the lateral PAG that are activated in response to female sexual behavior, as indicated by the co-expression of immediate early genes (Vaughn et al., 2022). This finding aligns with previous research demonstrating Fos expression in the lateral PAG following female sexual behavior (Yamada and Kawata, 2014). Dissecting the roles of these molecularly defined subpopulations within the PAG could provide deeper insights into their specific contributions to female sexual behavior. Given its position in the midbrain and proximity to motor output circuits, the PAG is considered a pivotal output center for the modulation of female sexual behavior. Further elucidation of the projections from PAG subpopulations to the downstream brainstem is crucial for a comprehensive understanding of the neural circuits that govern female sexual behavior.

The role of the medial preoptic area (mPOA) in female sexual behavior is complex and subject to ongoing debate. Electrical stimulation or lesion of the mPOA change the behavior facilitatory and inhibitory directions, indicating its nuanced role in modulating the behavior (Moss et al., 1974; Bast et al., 1987; Takeo et al., 1993). Studies utilizing single-unit recording and fiber photometry imaging techniques have observed that neurons within the mPOA are particularly active during instances when a female receives male mounting, with their activity diminishing as the male dismounts (Aou et al., 1988; Sakuma, 1994; Kato and Sakuma, 2000; Wei et al., 2018). Consistent with these findings, recent fiber photometry imaging of the activity of hormone-sensitive Esr1^+^ mPOA neurons revealed that they are similarly active when a female receives male mounting (Wei et al., 2018). A two-photon calcium imaging study demonstrates the role of Neurotensin^+^ mPOA neurons (84% of them are Esr1^+^) in the initial stages of sexual behavior. These neurons activate when a female encounters male pheromones, and optogenetic suppression of their activity results in a decreased attraction towards males, indicating a role in the pre-copulatory phase of sexual behavior (McHenry et al., 2017). These findings suggest the critical roles that genetically identified neuronal populations in the mPOA play in orchestrating female sexual behavior. However, causal relationships of these populations in copulation remained elusive. Recently, Ishii et al. (2023) discovered that a subset of GABAergic neurons in the mPOA reacts to the completion of mating, specifically when a female receives male ejaculation. These neurons were identified and labeled separately from those activated by pre-mating attractive behavior, allowing for targeted investigation into their respective roles in modulating female sexual behavior. Chemogenetic activation of the mating completion-activated neurons, but not those activated by attractive behavior, led to a reduction in female sexual receptivity. This finding suggests the presence of an mPOA neuronal subpopulation capable of suppressing sexual behavior, highlighting a complex regulatory mechanism within the mPOA. Further complexity will be revealed in the nature of Esr1^+^ mPOA neurons, the majority of which are GABAergic, suggesting an interplay between estrogen signaling and inhibitory neurotransmission within this brain region (Wei et al., 2018; Knoedler et al., 2022). RNA sequencing studies have identified diverse clusters of neurons within the mPOA, pointing to a rich mosaic of functional and phenotypic heterogeneity (Moffitt et al., 2018; Knoedler et al., 2022). Future studies into these subpopulations will unravel the detailed mechanisms by which the mPOA influences both the attraction phase and the copulatory process in female sexual behavior, offering deeper insights into the neural underpinnings of these complex behaviors.

Another hypothalamic region which is important for female sexual behavior is the anteroventral periventricular nucleus (AVPV). This region is also rich in expression of estrogen and progesterone receptors (Xu et al., 2012; Yang et al., 2013). Recent research has shown that ablation of a subset of AVPV neurons, kisspeptin^+^ AVPV neurons, leads to a reduction in female sexual receptivity, whereas optogenetic activation of these neurons enhances the behavior (Hellier et al., 2018). PR^+^ VMHvl neurons exhibit structural plasticity depending on sex hormones (Inoue et al., 2019). Labeling projections of PR^+^ VMHvl neurons with virally-encoded mCherry fused to the synaptic vesicle synaptophysin (Syp:mCherry) revealed that there is a dramatic, 3-fold increase of presynaptic termini in the AVPV in OVX primed and natural estrus females. Most of PR^+^ VMHvl neurons are glutamatergic. The amplitude of optogenetically evoked EPSCs in AVPV neurons, following ChR2-assisted stimulation of PR^+^ VMHvl neuronal axon termini, is larger in OVX primed females compared to OVX females. Subsequent experiments utilizing an optogenetic approach have emphasized the pivotal role of this PR^+^ VMHvl to AVPV circuit in controlling female sexual receptivity. These findings suggest that sex hormones enhance excitatory synaptic transmission through an increase in glutamatergic projections within PR^+^ VMHvl to AVPV circuit. Ovarian hormones induce plastic changes in many brain regions (Inoue, 2022). This study represents the first to link hormone-induced plasticity with behavioral alterations throughout the estrous cycle, offering insights into the neural mechanisms underpinning female sexual behavior. As described above, Cckar^+^ VMHvl neurons are a subset of PR^+^ VMHvl neurons critical to regulate female sexual behavior. Because almost all of PR^+^ VMHvl neurons are glutamatergic, Cckar^+^ VMHvl neurons are also glutamatergic. These Cckar^+^ VMHvl neurons also project to the AVPV, mPOA, and PAG. Retrograde labeling of AVPV-projecting neurons combined with cell body labeling of Cckar^+^ VMHvl neurons revealed that over 70% of AVPV-projecting VMHvl neurons are Cckar^+^. In addition, the same as PR^+^ VMHvl neurons, Cckar^+^ VMHvl neurons also increase their presynaptic termini, labeled with Syp:mCherry, in OVX primed females, while Cckar-VMHvl neurons do not (Knoedler et al., 2022). Thus, Cckar^+^ VMHvl neurons exhibit preferential projection to the AVPV and structural plasticity, suggesting that the Cckar^+^ projection within PR^+^ VMHvl to AVPV circuit plays a crucial role in controlling female sexual behavior.

As the VMH, mPOA, AVPV, and PAG are interconnected to each other, how information processing is achieved in these neural circuits is an important future question to understand the whole picture of the neural circuit mechanisms controlling female sexual behavior.

## Discussion

Classic studies have identified the pivotal roles of sex hormones and specific brain regions in regulating female sexual behavior. Recent advancements, including transcriptomic analyses and the viral delivery of transgenes to molecularly identified neuronal populations, have enhanced our understanding of how these populations govern behavior. Future research should aim to further elucidate the mechanisms through which sex hormone-mediated regulation of neural circuit functions and behaviors occurs. A critical avenue of investigation involves deciphering the molecular underpinnings of hormone-dependent neural plasticity. Estrogen signaling is important for the structural plasticity in many brain regions including PR^+^ VMHvl neurons. Utilizing data from recent transcriptomic analyses will be essential in identifying the genes responsible for such structural plasticity. Intriguingly, male PR^+^ VMHvl neurons do not exhibit the same increase in presynaptic termini as their female counterparts, while the most of PR^+^ VMHvl neurons express Esr1 in both sexes (Inoue et al., 2019). It is likely that the ability of PR^+^ VMHvl neurons to rewire the circuit in adults is developmentally hard-wired into the female brain. Such sexually dimorphic structural plasticity could be a result of difference in gene expression downstream of Esr1. Examining differences in Esr1-mediated gene expression between sexes could shed light on the mechanisms behind this sexually dimorphic structural plasticity. Further studies demonstrated that VMHvl neurons change their firing activity across the estrous cycle, and this may also result in cyclic change in female sexual behavior across the estrous cycle (Yin et al., 2022). Identifying the genes whose expression changes in response to hormonal fluctuations within these populations could provide insights into the nature of these plastic changes.

Characterizing the entire neural circuits controlling female sexual behavior in the estrous cycle-dependent manner is another important direction. The major outputs of VMH neurons are to the AVPV, mPOA, and PAG. The VMH to PAG circuit has been considered essential for initiating female sexual behavior, largely because both regions contribute to the behavior and the PAG is more directly linked to motor control functions. However, a recent study has highlighted the significance of the PR^+^ VMHvl to AVPV circuit in female sexual behavior (Inoue et al., 2019). The AVPV is traditionally recognized for its role in fertility and the regulation of the estrous cycle itself. The involvement of AVPV neurons in modulating female sexual behavior has only recently been uncovered, indicating that previous studies may have overlooked the importance of the VMH to AVPV circuit (Hellier et al., 2018). The interconnected nature of the VMH, mPOA, and AVPV, along with the PAG’s receipt of outputs from these hypothalamic regions, underscores the complexity of the neural networks involved. A comprehensive examination of how information is processed within these interconnected circuits to facilitate female sexual behavior is necessary for a full understanding of the neural mechanisms at play. While not the focus of this review, it is important to note that olfactory cues play a crucial role in driving female sexual behavior (Fraser and Shah, 2014). VMH receives inputs from circuits engaged in pheromonal information processing such as the medial amygdala (MeA) and bed nucleus stria terminalis (BNST) (Lo et al., 2019). Research has shown that the MeA, but not the BNST, is involved in pheromone-induced female sexual behavior (Ishii et al., 2017). The application of recent technical advancements, like multi-fiber photometry, allows for the simultaneous recording of activity across various brain regions (Kim et al., 2016; Guo et al., 2023). This, combined with epistasis behavioral experiments that can either suppress or activate specific nodes within the circuits, offers to dissect the full scope of neural circuits and their relationships in controlling female sexual behavior.

Lastly, exploring beyond the neural circuits that control female sexual behavior to include those governing rejection and mating completion behaviors is essential for a comprehensive understanding of reproductive strategy. Rejection behavior serves to avoid non-productive mating, optimizing energy efficiency and minimizing predation risk. During diestrus, when circulating ovarian hormone levels are low, females actively reject male mounts. This raises the question: Is rejection behavior merely a suppressed form of sexual behavior, or does it involve distinct neural circuits specifically for rejection? As described in the previous section, a recent study of anterior VMHvl controlling rejection suggests the latter possibility (Gutierrez-Castellanos et al., 2023). Another finding from different group also indicates that BNST is important for pheromone-induced rejection behavior (Osakada et al., 2018). One possibility is that the neural circuitry for female sexual behavior and rejection operates in a reciprocal inhibitory manner, modulated by ovarian hormone levels, to produce varied behaviors throughout the estrous cycle. Understanding the intricate connections between these neural circuits is crucial for elucidating how these diverse behaviors are orchestrated across the estrous cycle. Mating completion, characterized by a female receiving male ejaculation, triggers a state of satiation that diminishes sexual behavior (Zhou et al., 2023). Similar to rejection, mating completion leads to behaviors that avoid further male interaction, such as decreased attraction and reduced likelihood of copulation. However, mating completion should be seen as a state leading to gestation rather than an isolated behavior, suggesting that the neural circuits involved in mating completion may differ from or be upstream of those controlling rejection. Investigating the activity dynamics of neural populations responsible for rejection during mating completion, and vice versa, could offer valuable insights into the similarities and differences between these behaviors and states. As described, a subpopulation of mPOA neurons has been identified to encode mating completion (Ishii et al., 2023). Interestingly, another subpopulation of mPOA neurons, Galanin^+^ mPOA neurons, undergo significant plastic changes during pregnancy, contributing to parenting behavior post-delivery (Ammari et al., 2023). Analyzing the overlap or interconnectivity between neurons involved in mating completion and Galanin^+^ mPOA neurons could provide deeper understanding into the post-mating completion processes essential for successful reproduction. This comprehensive approach to studying the neural underpinnings of female sexual behavior, along with rejection and mating completion, is pivotal in unraveling the complex interplay of behaviors associated with reproduction.

Females, including women, exhibit various behavioral changes throughout their cycle. The dramatic fluctuations in rodent female sexual behavior across the estrous cycle, influenced by ovarian hormone levels, serve as an excellent model for exploring how ovarian hormones modulate neural circuits and, consequently, behaviors. Insights gained from such research can significantly contribute to our understanding of the etiology of women’s psychiatric disorders, including premenstrual dysphoric disorder, postpartum depression, and symptoms associated with menopausal syndrome. By studying these hormonal modulations and their effects on behavior, we can deepen our understanding of these complex conditions and advance towards more effective treatments.

## Author contributions

SI: Writing – original draft, Writing – review & editing.
